# Research on the association between beverages consumption and cancer

**DOI:** 10.3389/fmed.2025.1624496

**Published:** 2025-08-01

**Authors:** Alapati Waili, Shayidan Abdunayim, Maidina Talifu, Zhaoxin Zhuo, Maidina Ruzimaimaiti, Liwen Tao, Wei Han

**Affiliations:** ^1^Department of Pancreatic Surgery, First Affiliated Hospital of Xinjiang Medical University, Urumqi, China; ^2^Department of Clinical Medicine, Xinjiang Medical University, Urumqi, China; ^3^Department of Obstetrics and Gynecology, First Affiliated Hospital of Xinjiang Medical University, Urumqi, China

**Keywords:** cancer, beverages, cancer risk, bibliometrics, WOSCC

## Abstract

**Background:**

Cancer is the second leading cause of global death in the 21st century. Despite international efforts such as the European Code Against Cancer and the World Health Organization’s Global Action Plan, there are still significant gaps in translating policies into tangible outcomes. Diet, especially sugar intake, is a key modifiable factor affecting cancer risk, and the consumption of various beverages is associated with cancer susceptibility. This study uses bibliometric methods to analyze the research on the relationship between beverage consumption and cancer, aiming to provide a macro perspective and guide future research.

**Methods:**

We searched for articles related to beverage consumption and cancer published between January 2005 and January 2025. Utilizing bibliometric methods, we analyzed the publications using R software, VOSviewer, and CiteSpace. Our analysis encompassed an examination of the publication volume, citation behavior, and co-authorship networks, aiming to reveal the research dynamics and trends within the field.

**Results:**

We conducted a bibliometric analysis of 998 articles and found that the number of publications related to beverage consumption and cancer has been increasing steadily year by year. Among many countries, the United States and China are the main contributors in this field. Among the top 10 institutions, the National Cancer Institute has published the most research results, with a total of 111 papers, indicating its significant influence in this research area. The citation bursts of keywords reveal that the current and future research focus is on exploring the complex relationship between specific dietary factors and various types of cancer.

**Conclusion:**

Existing research indicates that over the past 20 years, there has been a steady increase in the number of research papers on beverage consumption and cancer. In this study, we conducted a comprehensive and impartial analysis of the relevant literature available in the WoSCC. We utilized various software tools to perform quantitative and visual analyses, such as examining the number of publications, citation counts, countries, institutions, journals, authors, cooperative relationships, and keywords. These findings enable researchers to identify emerging topics and frontiers in the field of beverage consumption and cancer, providing scholars with valuable insights and references.

## 1 Introduction

As a formidable global health challenge second only to cardiovascular diseases in both mortality and incidence rates, malignant neoplasms accounted for 16.8% of worldwide deaths in the 21st century (1). Contemporary epidemiological analyses underscore the escalating global cancer burden, with 2022 surveillance data indicating an estimated 20 million incident cases of malignant neoplasms worldwide. This disease panorama is further complicated by 9.7 million annual cancer-specific deaths, equivalent to 1 in 6 mortality events globally. Longitudinal projections demonstrate significant oncological risks across populations: cumulative lifetime incidence approximates 19.3% for males and 18.4% for females, while sex-stratified mortality probabilities reach 11.2 and 8.3%, respectively (2). The 4th edition of the European Code Against Cancer (ECAC) establishes 12 evidence-based recommendations for population-level cancer prevention across EU member states, concurrently outlining ambitious global targets for cancer burden mitigation (3). This initiative demonstrates remarkable synergy with the WHO’s 2013–2020 Global Action Plan for the Prevention and Control of Non-communicable Diseases (NCD), which mandates a 25% relative reduction in premature mortality from four major NCD categories (cardiovascular pathologies, malignancies, diabetes mellitus, and chronic respiratory disorders) by 2025 through multisectoral interventions (4). Despite these coordinated international efforts, significant implementation gaps remain in translating policy frameworks into tangible health outcomes, particularly in the context of cancer, a disease with numerous and complex risk factors. Therefore, over the past few decades, many researchers have been investigating the relationship between cancer and dietary habits, and exploring how changes in diet can reduce cancer incidence and mortality.

Diet serves as a pivotal modifiable determinant affecting cancer risk, therapeutic outcomes, and prognosis. The sugars present in food and beverages contribute to caloric surplus, thus high sugar consumption indirectly elevates cancer risk by promoting obesity (5). Emerging evidence has extensively investigated the epidemiological associations between various beverage consumptions (including coffee, sugar-sweetened beverages, carbonated drinks, and alcohol) and cancer susceptibility, with particular relevance to breast, colorectal, and hepatocellular carcinomas (6–8). Excessive added sugars in the American diet are a significant public health concern (9, 10). According to the 2020–2025 Dietary Guidelines for Americans (DGAs), added sugars should constitute less than 10% of daily caloric intake for individuals aged 2 years and older (11). However, population-level compliance remains suboptimal, as approximately 60% of U.S. adults exceed this recommended threshold, perpetuating obesity-related cancer risks (12). Various types of beverages have different compositions in terms of sugar, other nutrients, as well as calories, vitamins, minerals, food components, and other micronutrients, which may influence the risk of cancer (13, 14).

Although there is a substantial amount of literature on the correlation between beverage consumption and cancer, a comprehensive bibliometric analysis of this topic has not been conducted. Bibliometrics focuses on in-depth analysis of various aspects of scientific literature, including the total volume, quality, citation behavior, and interdisciplinary dissemination (15, 16). It helps to reveal key elements and knowledge structures in research, promoting a deeper understanding of the current state, research focuses, and future trends in a specific field (17, 18). Therefore, this study employs bibliometric methods, including R software, VOSviewer, and CiteSpace software, to analyze research on the relationship between beverage consumption and cancer. The aim is to provide a macro perspective on research in this field, reveal future development trends, and offer valuable insights and research directions for researchers. Through this approach, we can gain a more comprehensive understanding of the research dynamics in the field of beverage consumption and cancer, guiding future research and practice.

## 2 Materials and methods

### 2.1 Data acquisition and search strategy

Data were extracted from the Web of Science Core Collection (WOSCC) database, covering peer-reviewed articles and reviews published between January 2005 and January 2025.WoS is highly regarded for its authority and breadth in the field of academic information, encompassing over 12,000 core academic journals across various significant research areas. Due to its extensive coverage, WoS has become the preferred resource for bibliometric analysis (19). The search strategy utilized the following Boolean query to identify studies on beverage consumption and cancer:[TS = (“beverage*” OR “drink*” OR “soda” OR “soft drink*” OR “sugar-sweetened beverage*” OR “ssb” OR “fruit juice” OR “carbonated drink*” OR “coffee*” OR “sports drink*” OR “diet drink*” OR “low-calorie drink*” OR “unsweetened drink*”)] AND [TS = (“cancer” OR “tumor” OR “neoplasm” OR “malignancy” OR “carcinoma” OR “oncology” OR “breast cancer” OR “colorectal cancer” OR “gastric cancer” OR “prostate cancer” OR “pancreatic cancer” OR “liver cancer”)] NOT [TI = (“conference” OR “abstract” OR “editorial”)]. Filters were applied to include only English-language articles and reviews, excluding conference abstracts, editorials, and non-academic publications. The search strategy was validated using the PRESS Checklist to ensure sensitivity and specificity.

### 2.2 Exclusion criteria

**FIGURE 1:**
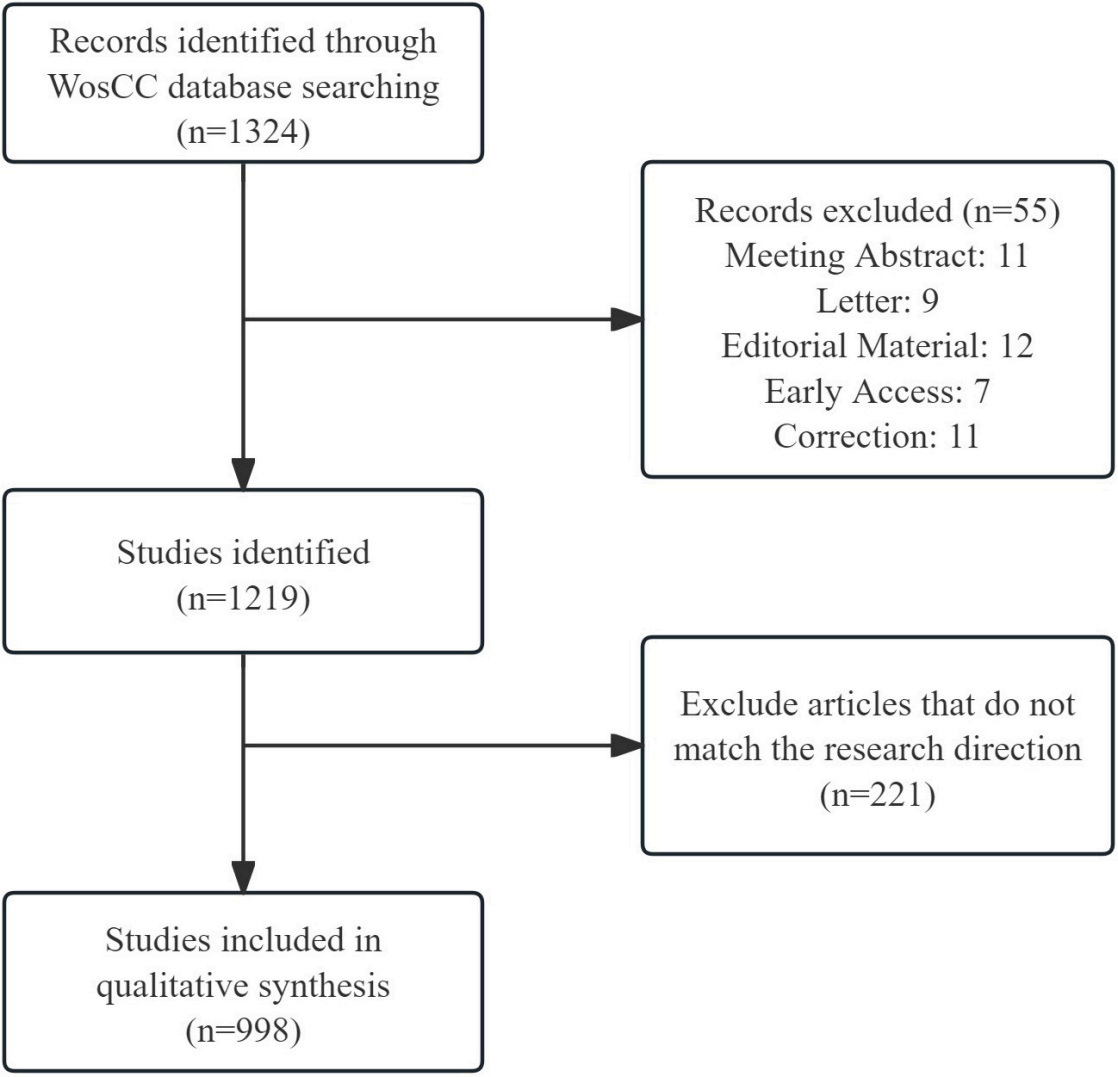
Flow chart of the data identification and screening results.

The study followed the updated methodological standards of the PRISMA 2020 framework (20) and excluded some studies via a two—stage screening process (Supplementary Data Sheet 1). In the automated screening phase, duplicates were removed through DOI and title matching, and non-research outputs such as conference proceedings and reports were excluded. In the subsequent manual screening phase, studies were excluded if they lacked quantitative exposure metrics or contained incomplete outcome data. Discrepancies in study inclusion were resolved through consensus between two independent reviewers. Ultimately, the study identified 998 publications related to beverage consumption and cancer (Figure 1).

### 2.3 Data analysis and visual network analysis

To conduct a comprehensive bibliometric analysis of beverage consumption and cancer research, we employed three analytical tools: R (v4.4.1), VOSviewer (v1.6.20), and CiteSpace (v6.4.R1). VOSviewer, developed by Van Eck and Waltman at Leiden University, specializes in constructing and visualizing large-scale bibliometric networks (21, 22). This study utilized VOSviewer to generate co-occurrence networks of keywords and institutions. Key parameters included a full counting method for term frequency, and cluster resolution optimization to delineate thematic patterns. Temporal overlay maps were created to trace evolutionary research trends. CiteSpace, a tool designed for detecting emerging trends and critical transitions in scientific literature, was applied to identify citation bursts and pivotal concepts (23, 24). The analysis, conducted annually from 2005 to 2025, used Pathfinder network pruning to simplify connectivity. Burst detection analysis, which calculates burst strength via Kleinberg’s algorithm to identify periods of unusually high keyword frequency in an event stream, was performed to spot keywords with sudden attention increases (25). A higher burst—strength value means the keyword’s usage surged morely and abruptly in scientific literature during the specific burst period. R software (v4.4.1) with the bibliometrix package facilitated advanced statistical evaluations (18).

## 3 Results

### 3.1 Overview

**FIGURE 2:**
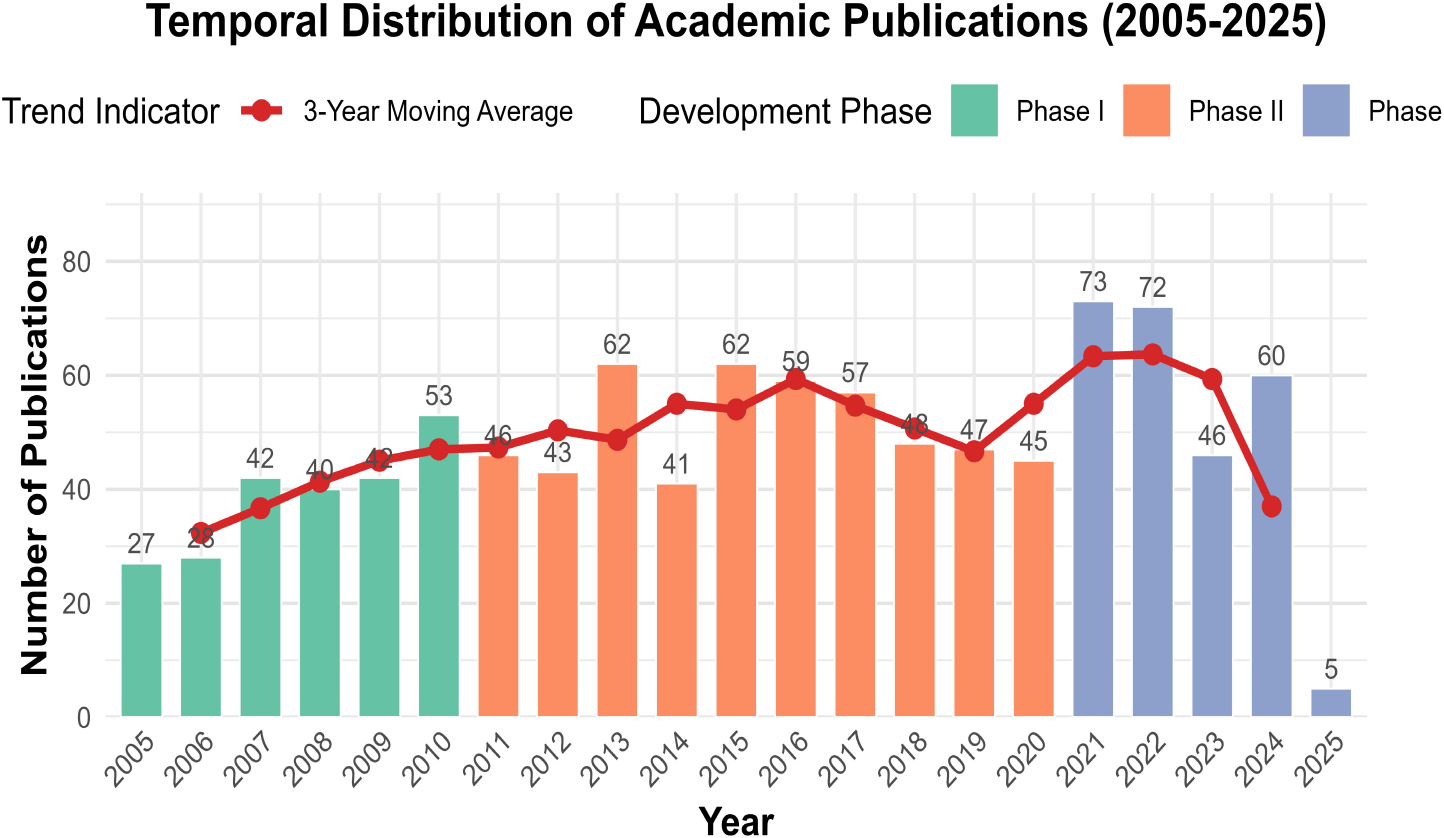
The annual number of publications.

In this study, we included a total of 998 academic articles related to beverage consumption and cancer. Overall, from 2005 to 2025, there was a stable upward trend in the number of publications in this research field (Figure 2). We used a trichromatic scheme to divide the time span into three developmental stages. Stage 1 (2005–2010): It was the initial growth period (*M* = 38.4, SD = 6.7). Stage 2 (2011–2020): It marked the stable development stage (*M* = 48.6, SD = 7.2). Stage 3 (2021–2025): It reflected the recent dynamics (*M* = 59.2, SD = 23.9). With the help of a 3-year moving average (red line), short-term fluctuations were smoothed out, revealing a continuous upward trajectory from 2005 to 2013 (Slope = 4.2 publication/year). From 2014 to 2020, a plateau was formed (an increase of 0.8 publications per year). The fluctuations after 2020 indicated a paradigm shift. It should be emphasized that in 2021, the publication volume reached its peak (*n* = 73), which was closely related to the emerging research hotspots and the increasing attention to the relationship between beverage consumption and cancer. In addition, the high standard deviation in Stage 3 also suggested that the research in this field was becoming more diversified and in-depth.

### 3.2 Country and regional publication analysis

**FIGURE 3:**
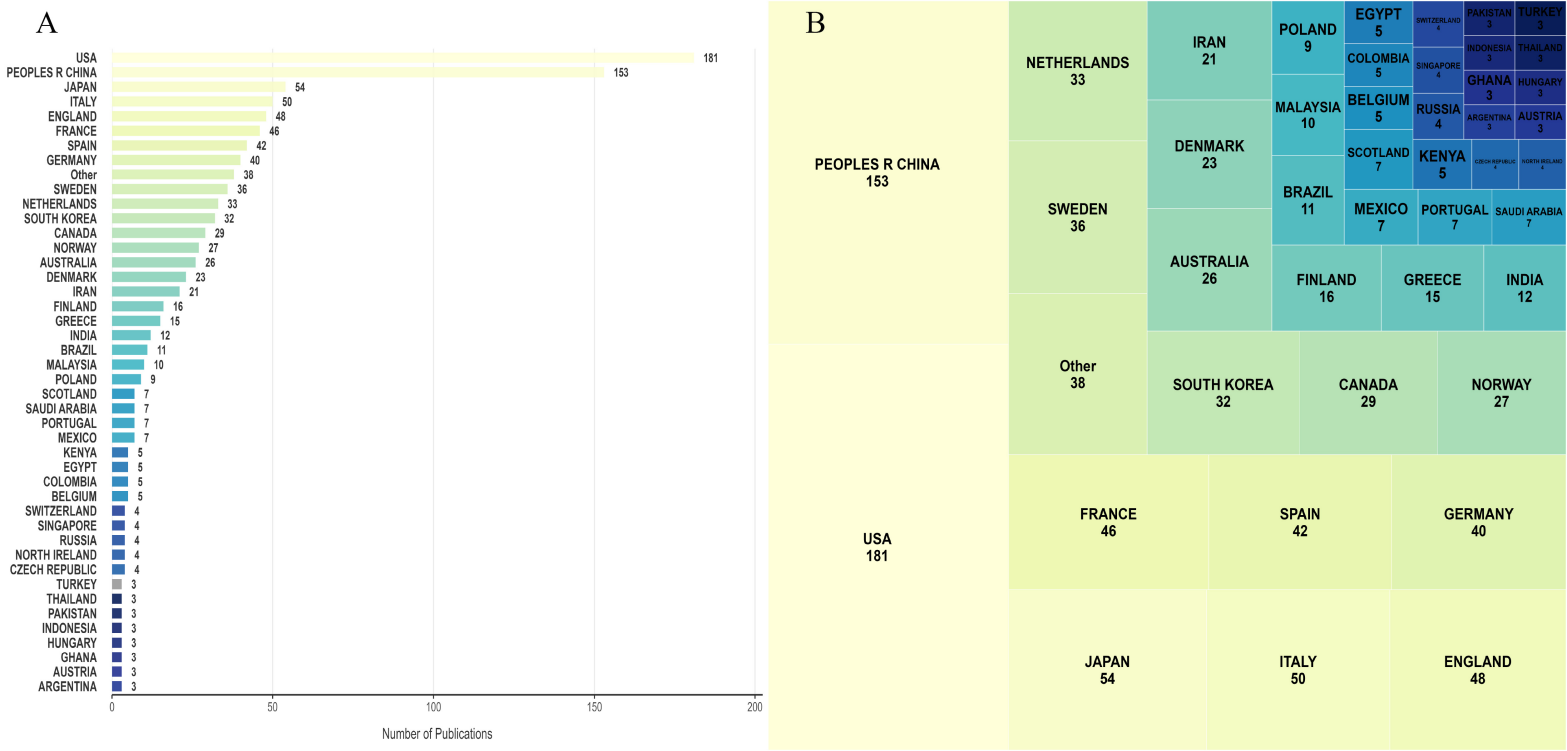
Countries and the number of articles published. **(A)** Bar chart of publication output by country; **(B)** tree diagram of publication output by country.

A total of 998 research papers on the topic of beverage consumption and cancer have been published, covering 78 different countries (Figure 3 and Table 1). The majority of these papers originated from the United States and China, with significant contributions also coming from Japan, Italy, and the United Kingdom. It is emphasized that the United States ranks first in the total citation index with 15,661 citations, while China follows with 6,375 citations, highlighting the prominent positions of both countries in the field of beverage consumption and cancer research.

**TABLE 1:** Top 10 countries with most publications.

Rank	Country	Documents	Citations	Average citations
1	USA	328	15,661	47.72
2	People’s R China	255	6,375	25.00
3	Japan	121	4,083	33.74
4	Italy	102	6,254	61.31
5	France	96	5,965	62.14
6	England	80	4,144	51.80
7	Sweden	77	3,070	39.87
8	Spain	61	2,783	45.62
9	Netherlands	58	1,821	31.39
10	Germany	54	1,671	30.94

We conducted bibliometric research to explore the cooperation between countries and regions. Italy and France had the most frequent cooperation, reaching 50 times, followed by the United States and China, with 39 instances of collaboration. In order to gain a deeper understanding of the extent of cooperation among these 78 countries, we carried out a co-authorship analysis.

**FIGURE 4:**
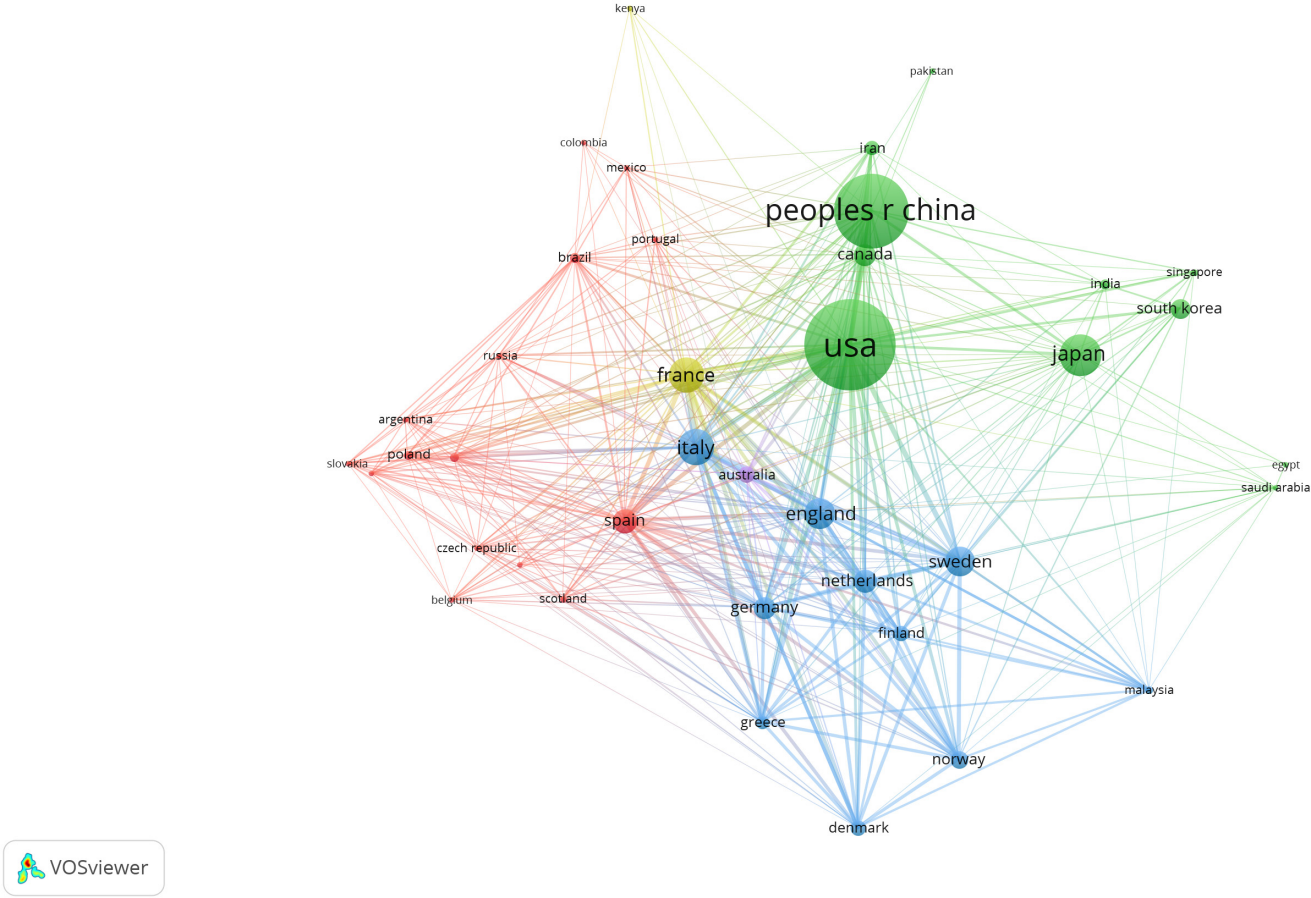
Network clustering of country co-authorship analysis.

The clustering network intuitively displays the number of publications through the size of the circles (Figure 4). The color of the circles represents the closeness of cooperation within the research team. Among them, the red cluster contains 15 items, the green cluster has 11 elements, and the blue cluster includes 10 elements. Argentina, Austria, Belgium, Brazil, Colombia, Czech Republic, Hungary, Mexico, Poland, Portugal, Russia, Scotland, Slovakia, Spain, and Switzerland belong to the red cluster; Canada, Egypt, India, Iran, Japan, Pakistan, China, Saudi Arabia, Singapore, South Korea, and the United States are in the green zone; Denmark, England, Finland, Germany, Greece, Italy, Malaysia, the Netherlands, Norway, and Sweden are grouped in the blue zone.

### 3.3 Institutional publication analysis

A total of 1,612 academic institutions have conducted research on the relationship between beverage consumption and cancer and published relevant results. Among them, 77 institutions have published at least eight articles. Table 2 the top 10 institutions based on the number of articles published. The National Cancer Institute has published the most research results, with a total of 111 papers. The Karolinska Institute and Harvard University have published 47 and 41 articles, respectively, following the top—ranked institution.

**TABLE 2:** The top 10 productive institutions/organizations.

Rank	Organizations	Documents	Citations	Average citations
1	National Cancer Institute	111	4,891	44.06
2	Karolinska Institute	47	2,179	55.98
3	Harvard University	41	2,631	64.17
4	Brigham and Women’s Hospital	39	2,211	56.69
5	University of Milan	39	3,867	99.15
6	Int Agcy Res Canc	36	3,687	102.42
7	Nagoya University	35	1,003	28.66
8	Harvard T.H. Chan School of Public Health	29	948	32.69
9	Tohoku University	28	910	32.50
10	Kochi Medical University	25	367	14.68

**FIGURE 5:**
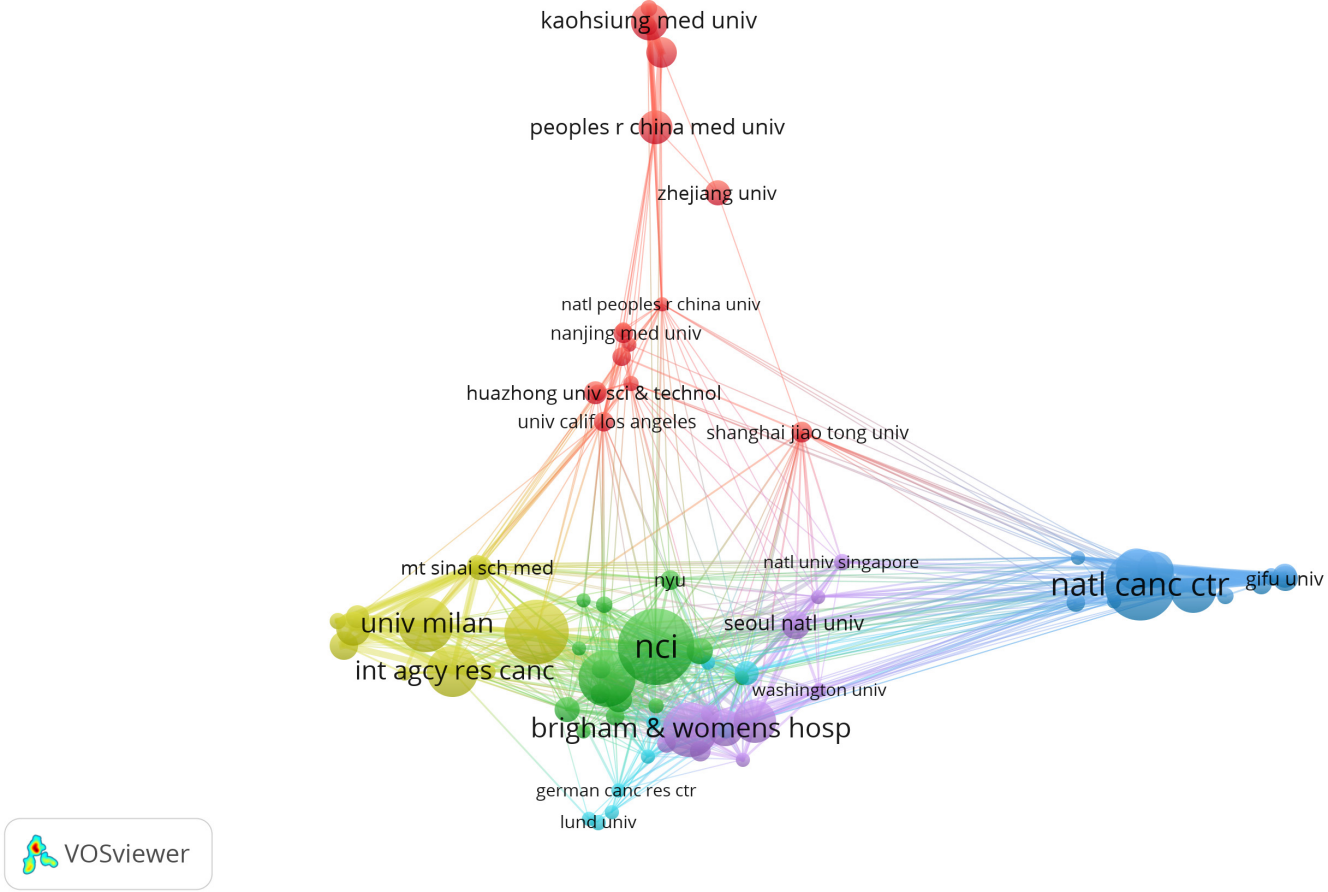
The co-authorship network of institutions.

We performed cluster analysis on these 77 institutions (Figure 5). The red cluster, mainly composed of 17 universities and institutions from China, is the largest one. The yellow cluster, which includes 15 universities and institutions such as Harvard University, is the second largest.

### 3.4 Analysis of journals

In this study, the articles have been disseminated across a wide range of academic journals, totaling 338 in number. Table 3 highlights the top 10 publications, presenting them along with their respective impact factors. Noteworthy half of these top 10 journals are in the JCR Q1 category, indicating their high academic standing. Among them, the “International Journal of Cancer,” “British Journal of Cancer,” and “American Journal of Clinical Nutrition” are particularly recognized as authoritative sources in the fields of cancer and nutrition research.

**TABLE 3:** The top 10 journals in terms of number of total publications.

Rank	Journals	Documents	Citations	IF/JCR
1	International Journal of Cancer	61	3,486	5.7/Q1
2	Cancer Epidemiology Biomarkers	40	1,603	3.7/Q2
3	Cancer Causes and Control	38	1,264	2.8/Q3
4	Nutrition and Cancer	27	611	2.7/Q1
5	British Journal of Cancer	25	840	6.4/Q1
6	Nutrients	22	402	4.8/Q1
7	European Journal of Cancer Prevention	21	681	2.1/Q2
8	Plos One	20	792	2.9/Q2
9	Scientific Reports	15	319	3.8/Q2
10	American Journal of Clinical Nutrition	12	755	6.5/Q1

**FIGURE 6:**
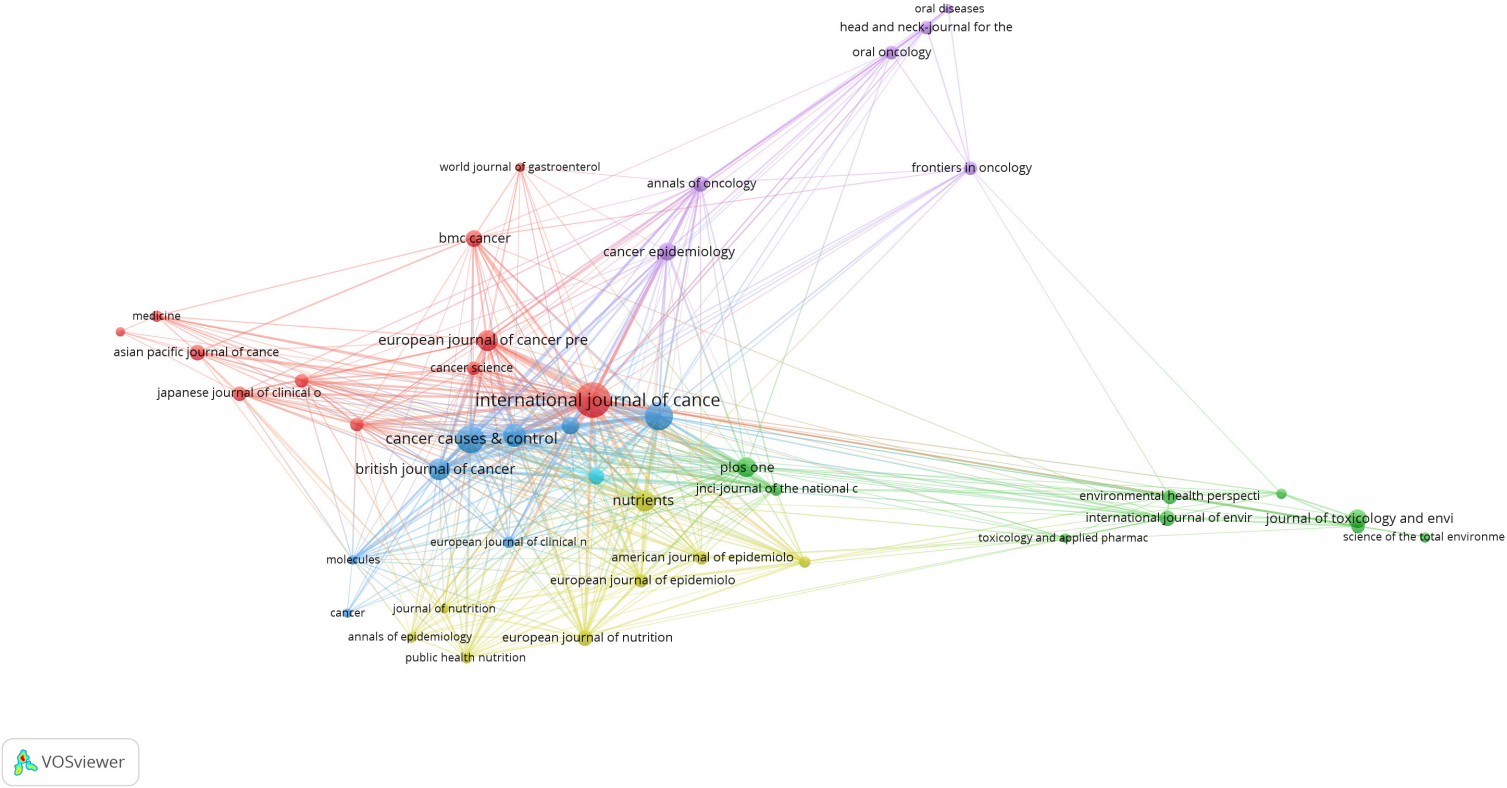
Journals with more than 5 publications network.

Furthermore, Figure 6 provides a network visualization of co-citation analysis, which was generated by VOS viewer. This visualization includes all journals that have been published at least five times, offering a clear picture of their interconnectedness. Within the 43 journals that met the criteria, some stand out in terms of co-citation frequency. The “International Journal of Cancer” (3,486 citations), leads with a significant number of citations, followed by “Cancer Epidemiology Biomarkers” (1,603 citations),“Annals of Oncology”(1,582 citations) and “Cancer Causes & Control” (1,264 citations). These citation statistics reflect the influence and importance of these journals in the relevant research domains, as they are frequently cited together by researchers, suggesting their interconnected roles in advancing knowledge in the field.

### 3.5 Authorship analysis

This study conducted a comprehensive review of the existing literature on the relationship between beverage consumption and cancer, aiming to delve into the research findings and academic contributions within this field. The analysis covered publications from 5,245 authors to assess their research activity and influence in this area. Among them, La Vecchia C, with 43 publications, was the most prolific author listed in Table 4, followed by Boffetta P, who published 28 articles, and Tsugane S, with 21 articles. Of these authors, 96 had published at least five related publications, indicating their continuous research and contributions to this field.

**TABLE 4:** The top 10 productive authors.

Rank	Authors	Documents	Citations	Average citations
1	La Vecchia C	43	1,687	39.23
2	Boffetta P	37	1,615	43.64
3	Tsugane S	39	936	24.00
4	Inoue M	35	866	24.74
5	Matsuo K	27	722	26.74
6	Tsuji I	22	590	26.81
7	Shimazu T	20	655	32.75
8	Wakai K	19	549	28.89
9	Wolk A	17	531	31.24
10	Giovannucci E	15	618	41.2

**FIGURE 7:**
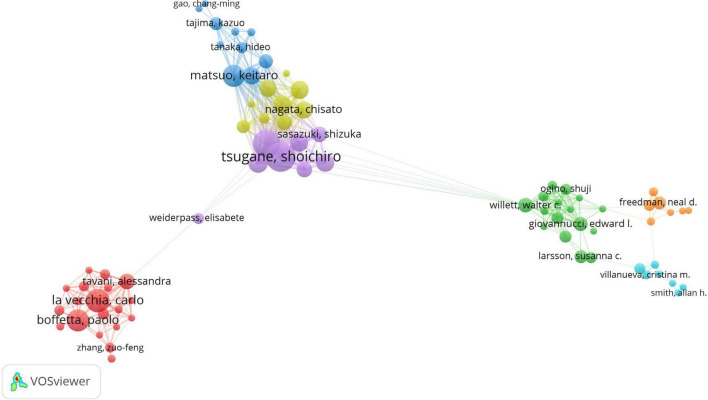
The co-authorship network of authors.

We conducted a network analysis on these 96 authors to reveal their collaborative relationships. In the network diagram, the size of the circles represented the number of publications for each author, while the colors were used to distinguish different clusters, and the lines between nodes indicated collaboration among authors. Figure 7 presents the results of this network analysis, showing that La Vecchia C, Tavani A, and Tsugane S had the highest frequency of collaboration. It is particularly worth noting that La Vecchia C had the largest node and the densest connections, with a collaboration frequency as high as 16 times, which not only demonstrated his significant academic contributions to this field but also reflected his extensive interactions and collaborations with other authors.

### 3.6 The analysis of keywords

**FIGURE 8:**
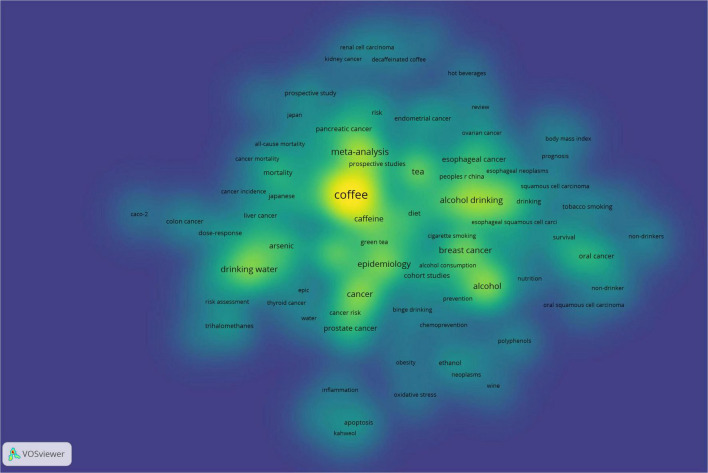
Density map of keywords.

Keyword co-occurrence analysis, a frequently employed bibliometric technique, illuminates the connections between research topics, academic disciplines, and concepts by examining the concurrent appearance of keywords within research literature (26). In this study, we analyzed 1,479 author keywords, of which 113 appeared at least five times. Through the creation of a density map, the distribution of prevalent themes across the field becomes readily apparent. As depicted in Figure 8, the most prominent elements are “coffee,” “alcohol consumption,” and “tea drinking,” which stand out distinctly on the density map.

**FIGURE 9:**
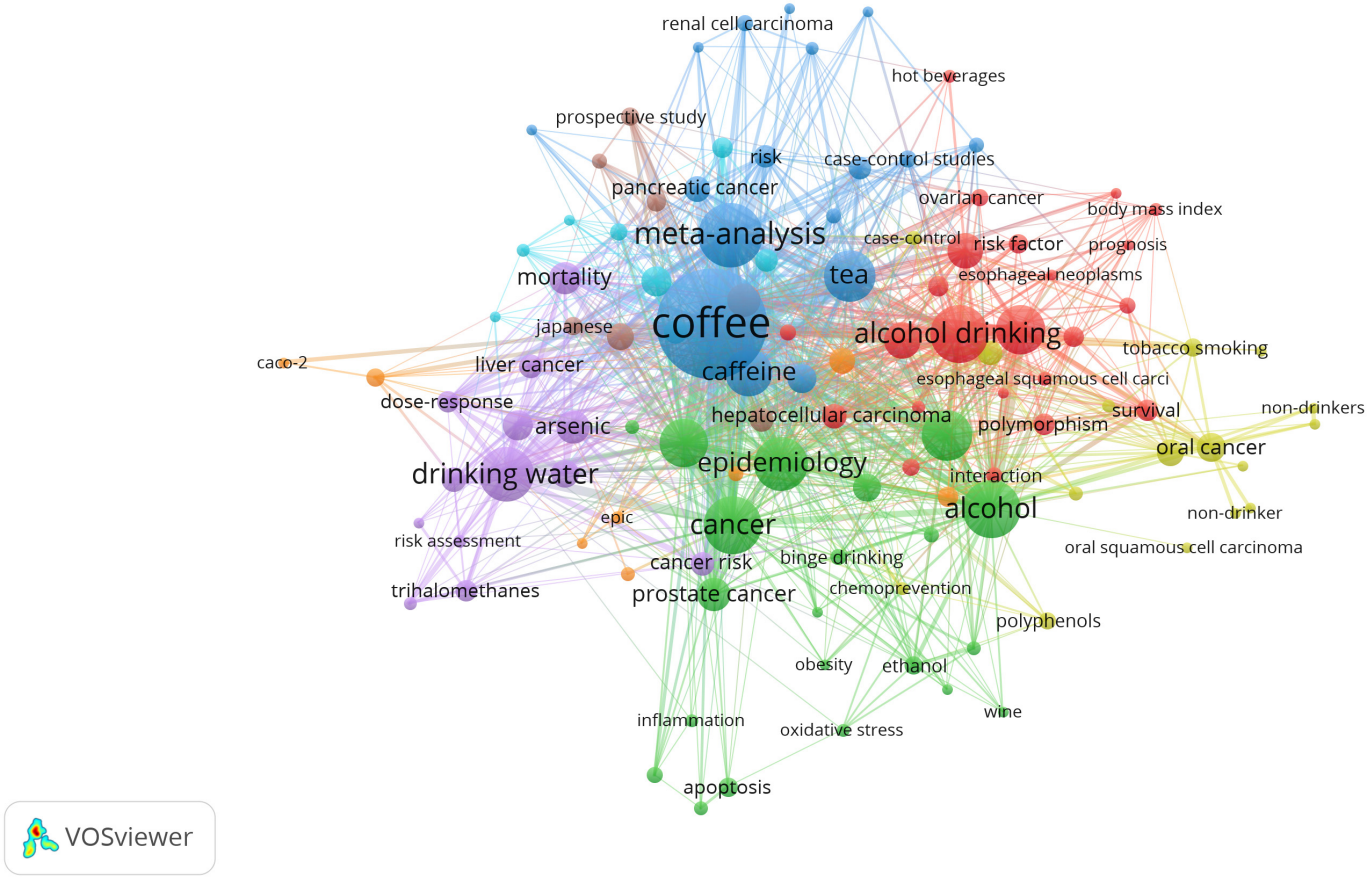
Network visualization of keywords.

**FIGURE 10:**
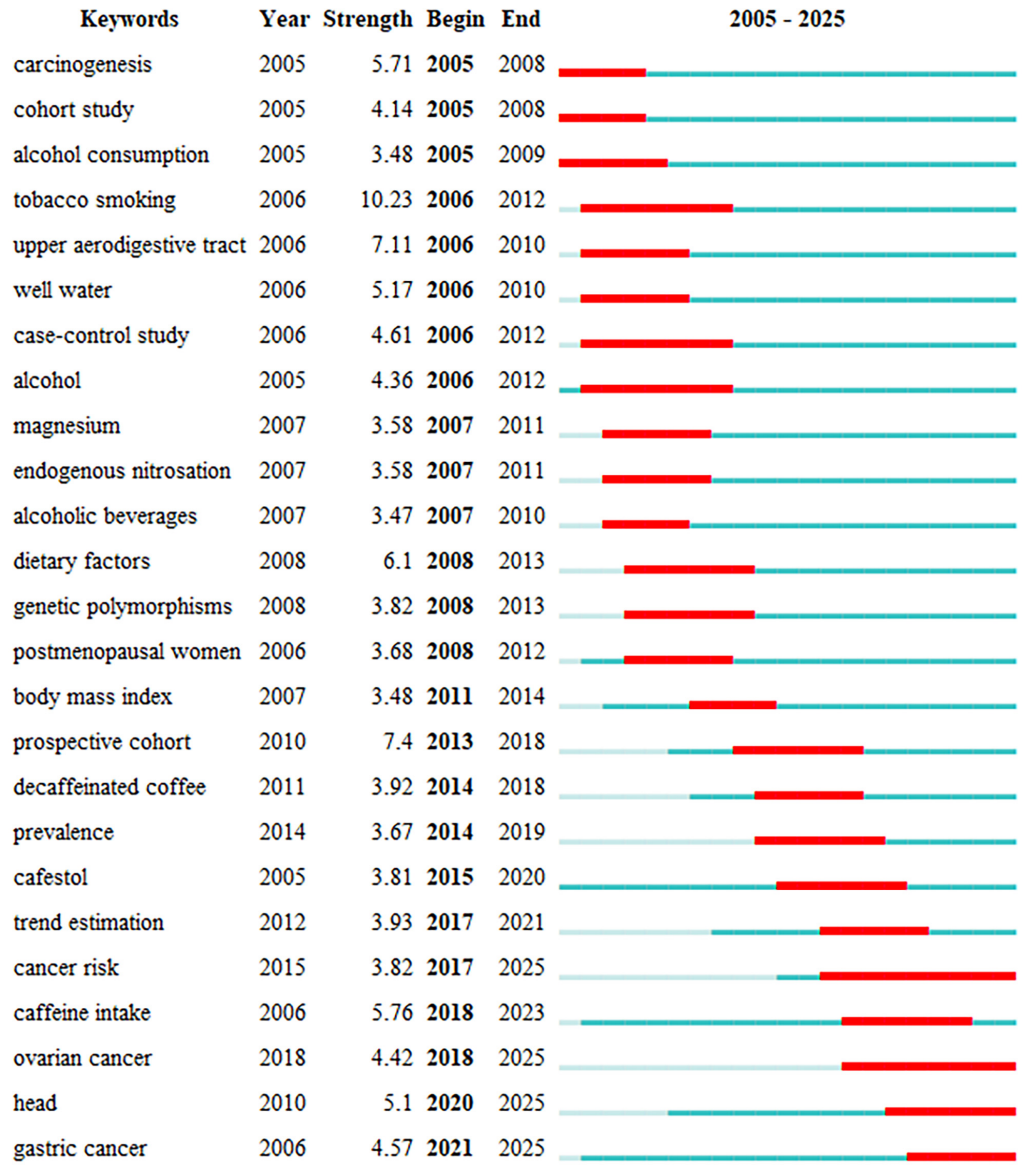
Visualization map of top 25 keywords with the strongest citation bursts.

We then conducted a cluster analysis using the selected keywords. Eight clusters, comprised of the 113 high-frequency keywords, represent the eight primary research areas within this topic (Figure 9). Cluster 1, the largest and marked by a red circle, primarily focuses on beverage types and includes a total of 29 keywords. Cluster 2, represented by a green circle, contains 21 keywords and mainly centers on mechanisms. Cluster 3, denoted by a dark blue circle, emphasizes the relationship between beverage types and various cancer types. The keyword burst detection analysis has revealed that certain keywords, such as “gastric cancer,” “cancer risk,” and “ovarian cancer,” have shown a continuous surge in research attention up to the year 2025 (Figure 10). This indicates a growing research focus on these specific cancer-related areas in the context of beverage consumption and its potential impact on cancer risk.

## 4 Discussion

This bibliometric analysis of 998 publications provides a comprehensive overview of global research trends in beverage consumption and cancer from 2005 to 2025. By integrating temporal, geographical, institutional, and thematic dimensions, our study reveals critical insights into the evolving scientific landscape, highlighting both progress and persistent gaps in this field. More importantly, this integrated bibliometric approach offers novel perspectives by uncovering distinct research phases, geographic clusters with specific thematic focuses, and the co-evolution of research priorities with methodological and policy advancements, providing a unique macro-level understanding beyond individual study findings.

### 4.1 Temporal trends and research evolution

The triphasic growth pattern of publication output—the initial growth phase (2005–2010), the stabilization phase (2011–2020), and the diversification phase (2021–2025)—is closely related to the key advancements in nutritional epidemiology and global cancer prevention policies. Between 2005 and 2010, the European Prospective Investigation into Cancer and Nutrition (EPIC) promoted the progress of large—scale cohort studies and provided foundational evidence for establishing the link between dietary patterns and cancer risk (27–30). In 2007, the WHO released its first report on diet and cancer prevention, which propelled research into the carcinogenicity of specific beverages (31, 32).

Between 2010 and 2020, the publication of relevant literature entered a period of stable development, which may be related to the standardization of dietary assessment methods, including the widespread adoption of metabolomic biomarkers such as using urinary sucrose as a proxy indicator for SSB intake, as well as the standardized food frequency questionnaire (FFQ) (33–35). It is worth noting that between 2014 and 2018, countries including Mexico and the UK implemented SSB taxation policies, which spurred policy—driven research on beverage consumption patterns (36–38). After 2020, relevant research entered a diversified development stage, and advances in nutrigenomics have made it possible to study the interaction between genes and beverages.

### 4.2 Geographical disparities: a reflection of resource allocation and disease burden

The findings of this study indicate that the United States and China are in a dominant position in the field of nutritional oncology, having published 181 and 153 articles respectively. This clearly reflects that the research investment of the two countries in this field is far greater than that of other countries. The leadership position of the United States in this field is mainly due to the continuous funding of $1.3 billion per year from the NIH since 2015 for research on diet and cancer (39–41); while the rapid rise of China in this field is in line with its “Healthy China 2030” agenda, which aims to deal with cancers related to lifestyle (42, 43).

The three geopolitical groups identified in this field include the European/Latin American group (red), the Asian/North American group (green), and the Northern European group (blue). These groups reveal the specific research focuses of each region, which are shaped by local dietary habits. The red group focuses on cancers related to alcohol. The study by Heath AK and others shows that higher alcohol intake is associated with a higher risk of breast cancer (hazard ratio (HR) = 1.05 for an increase of 1 standard deviation in intake, 95% CI 1.03–1.07), and this is also the case for beer/cider intake and wine intake (HR = 1.05 and 1.04 for each 1 standard deviation increase, 95% CI 1.03–1.06 and 1.02–1.06 respectively) (44). The green group emphasizes research on the link between SSB and cancer. A recent study on American adults has shown that there is a relationship between drinking SSB and the risk of colon cancer (HR = 1.33; 95% CI = 1.10–1.59). In addition, drinking SSB is also associated with an increased risk of severe chemotherapy—induced peripheral neuropathy in colon cancer patients (HR = 1.57; 95% CI = 1.14–2.18) (45). In the meantime, the study of McCullough et al. indicated that the consumption of SSBs was correlated with an increase in mortality from colorectal and kidney cancers (8). The blue group is leading in coffee research, which is consistent with the high coffee consumption in Northern European countries. These phenomena highlight the “localization paradox”: while research focuses in each region align with local consumption and disease burdens, this focus might conceal global issues. For instance, over—focusing on alcohol—related cancers in some groups may divert attention from the growing global burden of SSB—related cancers outside the green cluster and the under—studied potential protective effects of green tea, which is culturally significant in Asian countries. Our network analysis has quantified this geographical clustering bias, underscoring the need for more globally representative research agendas that go beyond local dietary patterns.

### 4.3 Institutional and journal analysis

The National Cancer Institute’s prominent position, with 111 publications, highlights its dual role in generating evidence and shaping U.S. dietary guidelines. A key point to emphasize most of the top institutions publishing research on beverage consumption and cancer are located in countries that have implemented SSB taxation policies. This suggests a feedback loop between research and policy implementation.

Regarding the journal analysis, the dominant position of the International Journal of Cancer and the American Journal of Clinical Nutrition in the co-citation network reflects their crucial role in bridging mechanistic studies and population—level research. However, the underrepresentation of journals focusing on low- and middle-income countries (LMICs) should be addressed. This imbalance may limit the generalizability of the research findings. To address this, we propose enhanced funding mechanisms for research led by LMICs through global health initiatives, capacity—building programs for local researchers, and ethical guidelines to promote equitable North–South research partnerships.

### 4.4 Analysis of keywords and research trends

The analysis of 1,479 author keywords revealed eight distinct clusters (Figures 8, 9), providing a granular view of the intellectual structure and evolving priorities in beverage—cancer research. These clusters span mechanistic and epidemiological, reflecting both persistent knowledge foundations and emerging frontiers.

Cluster 1 (Beverage—Specific Carcinogenicity, 29 keywords): This cluster focuses on the study of direct dietary carcinogens. Centered on “coffee,” “alcohol consumption,” and “sugar—sweetened beverages (SSBs),” it encapsulates decades of research into direct dietary carcinogens. The prominence of “coffee” aligns with its dual role. The meta—analysis by Turati F et al. indicated that coffee consumption has no significant association with pancreatic cancer risk, even at high intake levels (RR 1.03, 95% CI 0.99–1.06). Another study showed that compared with non-coffee drinkers, daily consumption of > 4 cups of caffeinated coffee is negatively associated with head and neck cancer (OR 0.83; 95% CI 0.69–1.00), oral cancer (OR 0.70; 95% CI 0.55–0.89), and oropharyngeal cancer (OR 0.78; 95% CI 0.61–0.99). The sustained focus on “alcohol” mirrors its classification as a Group 1 carcinogen for seven types of cancer, while “SSBs” emerged after 2010, correlating with the global obesity epidemic. There’s robust evidence showing SSBs boost weight gain, overweight and obesity risks, as proven by meta-analyses of RCTs and prospective cohort studies. Unlike calories in solid foods, SSB calorie intake isn’t offset by reduced consumption from other sources. Thus, SSB consumption may up cancer risk and mortality by causing obesity (46).

Cluster 2 (Molecular Mechanisms, 21 keywords): dominated by terms such as “oxidative stress,” “inflammation,” and “DNA damage,” this cluster reflects the field’s shift toward mechanism—driven research. For example, a recent study indicates that caffeine and other compounds in coffee, or factors linked to coffee drinking, might regulate K-Ras activation in cancers such as pancreatic cancer. This could happen by interfering with DNA repair, cell cycle checkpoints, and apoptosis (47).

Cluster 3 (Site—Specific Malignancies, 18 keywords): focusing on “colorectal cancer,” “breast cancer,” and “gastric cancer,” this cluster highlights cancer types with established dietary links. The surge in “gastric cancer” research from 2021 to 2025 parallels the rising global incidence linked to processed beverage additives.

The analysis of the strongest citation bursts of keywords indicates that in the early stage from 2005 to 2010, research was primarily focused on the mechanisms of carcinogenesis and the establishment of research methods. The citation bursts of keywords such as “carcinogenesis,” “cohort study,” and “alcohol consumption” show that the research focus at that time was on understanding the basic mechanisms of cancer formation and exploring the relationship between lifestyle factors and cancer risk through cohort studies. From 2011 to 2020, the research scope further expanded, with particular attention to specific dietary factors and their impact on cancer risk. Keywords such as “alcohol,” “magnesium,” and “endogenous nitrosation” continued to receive attention, indicating that research continued to delve into the role of specific nutrients and dietary components in cancer development. In recent years (2021–2025), research has increasingly focused on specific cancer types and their risk factors. The citation bursts of keywords such as “cancer risk,” “caffeine intake,” and “ovarian cancer” indicate that the current and future research focus is on understanding the complex relationship between specific dietary factors and various cancers. The continued importance of keywords such as “prospective cohort” and “trend estimation” shows that longitudinal studies and trend analysis will still play a key role in advancing knowledge in this field.

Overall, the research trend has evolved from basic research and the establishment of cohort studies to in-depth exploration of specific dietary factors, genetic polymorphisms, and cancer types. The early stage was dominated by basic research, the middle stage saw a broadening of research scope with a focus on specific dietary components and their interaction with genetic factors, and in recent years, there has been an increasing focus on specific cancer types and their risk factors, emphasizing the importance of precision nutrition and targeted interventions. Future research is expected to continue to explore the complex relationship between diet, lifestyle, and cancer risk, with a focus on developing personalized cancer prevention and treatment plans.

### 4.5 Practical recommendations for public health

In our study, we found that the link between certain beverage consumption and cancer risk may be a key focus of future research, such as the correlation between alcohol, coffee, sugary drinks and cancer risk. Given this, healthcare providers should include beverage intake assessment in routine cancer risk screening, highlight the connection between alcohol and other drinks with cancer, and offer personalized cancer risk reports based on patients’ beverage intake and health factors. This raises patients’ awareness of diet—related cancer risks, aids early risk identification, promotes healthier lifestyle choices, strengthens cancer prevention, and offers more targeted evidence for cancer prevention policies.

## 5 Limitations

This study has certain limitations that are associated with the methodological features of bibliometric research and the inherent constraints of the database used. Initially, while the WoS database boasts extensive coverage, it does not encompass all global documentary resources. Consequently, articles published in non-indexed journals, particularly those in non-core or emerging fields, may have eluded the scope of our analysis. This gap could compromise the comprehensiveness of our findings and may introduce bias in accurately reflecting the realities of diverse regions and research domains on a worldwide scale. Furthermore, the study’s language selection was limited to English-language articles. While this criterion facilitates unified data processing and analysis for the research team, it inevitably narrows the research perspective by excluding significant research findings from non-English-speaking countries and regions. The absence of these non-English works, which may offer unique viewpoints, innovative methodologies, or important data, could impose restrictions on the universality of the conclusions. As a result, the study’s findings may not fully represent the global research landscape.

## 6 Conclusion

This bibliometric analysis systematically examined 998 beverage—cancer studies (2005–2025). Three key development stages were identified, reflecting the driving force of scientific progress, method standardization, and policy intervention. Significant geographical disparities and a “localization paradox” were found: research focuses align with regional consumption and disease burdens but may overlook globally relevant issues, particularly the underrepresentation of LMICs. The rise of precision nutrition has made specific beverage-cancer links and their interactions with genetics and lifestyle the current research frontier. Future work should prioritize large-scale interdisciplinary studies to clarify gene-beverage-environment interactions for personalized risk prediction; enhance research in underrepresented areas to overcome the “localization paradox” and boost global applicability; strengthen LMIC research capacity and their presence in high—impact journals; and explore emerging beverage types and health effects. Addressing these priorities can offer more targeted public health strategies and dietary guidelines for global cancer prevention.

## Data Availability

The raw data supporting the conclusions of this article will be made available by the authors, without undue reservation.
